# The microbial etiology and resistance patterns of cosmetic tourism-related infections in Ireland

**DOI:** 10.1007/s11845-024-03800-8

**Published:** 2024-09-10

**Authors:** Andrew Keane, Aoife A. Feeley, Shu Ying Chee, Fiachra Sheil, Joanne O’Gorman, Eoghan O’Neill, Kevin C. Cahill

**Affiliations:** 1 Department of Surgery, Connolly Hospital Blanchardstown, Mill Rd, Abbotstown, D15 X40D Dublin Ireland; 2 Department of Plastic and Reconstructive Surgery, St James’s Hospital, James St, Saint James, D08 NHY1 Dublin 8 Ireland; 3 Department of Plastic and Reconstructive Surgery, Beaumont Hospital, Beaumont Road Dublin 9, Dublin, D09 V2N0 Ireland; 4 Department of Microbiology, Connolly Hospital Blanchardstown, Mill Rd, Abbotstown, D15 X40D Dublin Ireland

**Keywords:** Cosmetic tourism, Esthetic surgery, Plastic surgery, SSI

## Abstract

**Background:**

Cosmetic tourism is an increasingly common phenomenon, both in Ireland and internationally. Complications associated with procedures performed abroad may manifest after the patient has traveled back to their home country with patients often presenting to local health services. Such complications can be infectious in nature requiring either antimicrobial therapy and/or source control in the form of wound debridement or radiologically guided interventional procedures.

**Methods:**

Patients who had presented to a regional plastic surgery unit between September 2021 and December 2022 with complications related to aesthetic procedures performed outside Ireland were identified in this retrospective-prospective design. Medical records were reviewed to ascertain what procedures were performed, where the procedure had taken place, the nature of complications, and microbial culture and sensitivity results.

**Results:**

Thirty patients were identified during the study timeframe, predominantly female (*n* = 28) with a mean age of 40.27 years (SD 10.6). Abdominoplasty was the most common procedure (*n* = 17), and Turkey was the most frequently cited destination (70%). Wound dehiscence accounted for the majority of complications (*n* = 18). Wound cultures were obtained in 80% (*n* = 24) of patients and a causative organism was isolated in 85% (*n* = 34) of cultures. Eighteen species of bacteria were identified and antimicrobial sensitivities were obtained for 16 of these. Antimicrobial resistance to commonly prescribed empiric antibiotics was noted in several isolates.

**Conclusion:**

Post-operative infections related to cosmetic tourism are a growing concern in plastic surgery. The bacterial etiology is varied, and antimicrobial resistance poses significant challenges, highlighting the need for early intervention and wound cultures to guide effective management.

## Introduction

Cosmetic tourism is a term used to refer to patients traveling outside their home country for the express purpose of having an aesthetic procedure performed [1, 2]. This practice has been reportedly increasing both in Ireland [3, 4] and internationally [2, 5, 6] in recent years, although it is difficult to obtain accurate numbers—either of patients traveling or complications arising. Incentivizing factors are thought to be lower healthcare costs internationally, a desire for anonymity, or access to high-quality procedures otherwise unavailable in their home country [7–9]. Demand for elective cosmetic procedures is hindered by two factors limiting their supply, namely, the absence of a public service for cosmetic surgery and the limited capacity in the domestic private healthcare sector. This represents a further driver for patients to seek cosmetic treatments abroad. A burgeoning availability of private hospitals and clinics catering to cosmetic tourists exists in Europe and beyond. Popular destinations for cosmetic tourists from Ireland and the United Kingdom include Turkey and Lithuania [4, 10]. Institutions in such countries undertake advertising overseas in which procedures are priced competitively, often with flights and accommodation included [1–3]. Outside of Ireland, it may be unclear or difficult to check if the surgeon performing the procedure is a suitably credentialled plastic surgeon as there may not be a specialist register easily accessible to members of the public [11].

Cosmetic tourists often present with their complications to local health services in their home country due to limited post-operative care provided in the country of operation and the financial and logistical challenges related to returning to the hospital abroad [3, 6, 12]. Medical and consumer protection legislation may be disparate even between neighboring countries and patients may find they have limited recourse to return and receive further treatment or care in the case of complications [5]. Infection is among the most common post-operative complications encountered in cosmetic tourism, often felt to relate to inadequate sterilization and operative techniques [6], and presents as wound infection or dehiscence [3, 5]. Surgical site infections acquired from elective cosmetic procedures performed abroad present a challenge to domestic plastic surgery teams [8, 9].

Endemic microbiological flora can vary significantly from country to country, and cosmetic tourism may increase the potential for transmission of antimicrobial-resistant organisms [6, 13, 14]. Some organisms may be resistant to empirically prescribed antibiotics for similar domestic presentations and can lead to prolonged inpatient hospital stays and courses of treatment [15, 16]. Inadequate antibiotic stewardship, through inappropriate prescribing or non-prescription availability of drugs, in other countries can promote resistance [13]. In addition to this, rare species such as rapidly growing mycobacterium have been described in cosmetic tourists in several countries including the USA, Netherlands, and Lebanon [17]. However, limited data exist on the microbiological etiologies of patients presenting to plastic surgery units in Ireland with cosmetic tourism-related infections and the incidence of antimicrobial resistance in this population.

## Methods

We designed a retrospective-prospective cohort study that identified patients presenting to our regional plastic surgery unit due to infections relating to cosmetic procedures performed abroad between September 2021 and December 2022. This was prompted when we noted an increase in these cases presenting to our service. Ethical approval for this study was granted by the hospital research and ethics committee. The patients included in our study were identified through review of trauma clinic and theatre logbooks and prospectively maintained for the stated study period. Once identified, the medical records of each patient were then reviewed and the following data points were extracted: demographic details (including age, gender, and county of residence), type of cosmetic procedure performed, country where cosmetic procedure was performed, nature of complication encountered, microbial culture swabs taken, site of culture, organisms isolated on microbial cultures, antimicrobial susceptibility or resistance of isolates, type and number of source control interventions required, the need for admission to hospital, and number of inpatient days required. Descriptive analysis of demographic data, microbial etiology of complications, microbial culture results, and antimicrobial susceptibility was performed. Inferential analysis carried out with Fisher’s exact test to evaluate the relationship between microbiology cultures and country of procedure was performed with a type 1 error rate of 0.05 set.

## Results

### Demographics

**Fig. 1:**
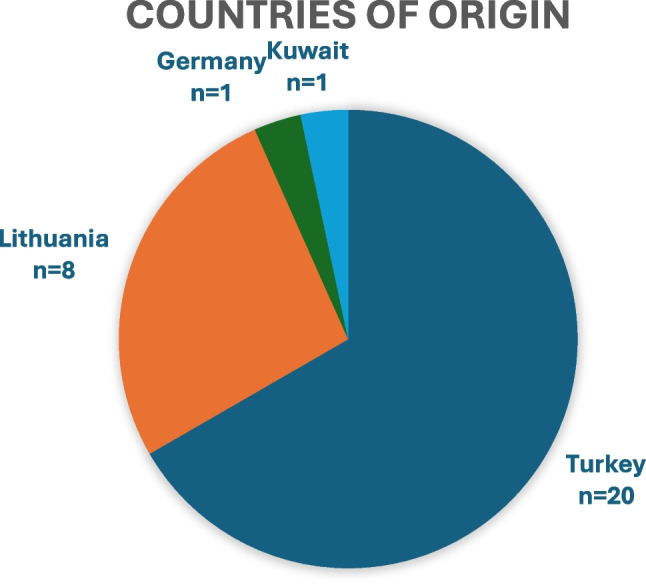
Distribution of countries where cosmetic procedures performed

Our study identified 30 patients who were seen by our department due to complications from cosmetic tourism during the 15-month study time frame. Of these patients, 93.3% were female (*n* = 28), 6.6% were male (*n* = 2), and the mean age was 40.54 years (SD 10.8). The geographic distribution of original procedures is illustrated in Fig. 1, with Turkey being the most common destination (*n* = 20), followed by Lithuania (*n* = 8), while Germany and Kuwait each accounted for a single patient. Twelve patients (40%) had multiple procedures combined into a single surgery.

### Procedural types

**Fig. 2:**
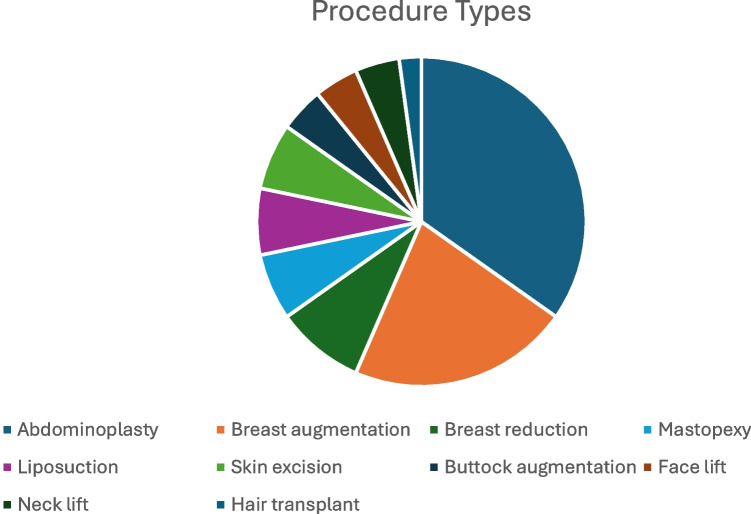
Types of cosmetic procedure performed

Abdominoplasty was the most common operation encountered (*n* = 16) followed by breast augmentation (*n* = 10), with breast reduction (*n* = 4), mastopexy (*n* = 3), liposuction (*n* = 3), skin excision (*n* = 3), buttock augmentation (*n* = 2), face lift (*n* = 2), neck lift (*n* = 2), and hair transplantation (*n* = 1) also referred (Fig. 2). Fourteen patients underwent a single procedure, 3 patients underwent 2 procedures, and 5 patients underwent 3 procedures.

### Infective complications

The infectious complications encountered (Table 2) were classified according to the Centers for Disease Control and Prevention (CDC) guidelines on Surgical Site Infection (SSI) [18]. All infections in our population constituted superficial incisional SSIs, occurring within 30 days of the index procedure, and involved the skin and subcutaneous tissue. Wound dehiscence secondary to infection was the most common presentation (*n* = 22) followed by seroma (*n* = 6); skin necrosis (*n* = 2) and infected hematoma (*n* = 1) accounting for the remainder of cases.

### Infectious etiology

A likely microbial etiology was demonstrated in 18 of the 30 patients included in this study. Of our 30 patients, 80% (*n* = 24) had tissue, pus, fluid, or wound swabs sent for microbial culture with 40 specimens being recorded in total. Considerable variation in both quality and timing of samples was noted with some being obtained on admission, others during inpatient stay and several in the course of surgical intervention. Admission screening was conducted for CPE in 70% and MRSA in 60% of cases with no positive screens detected. Discounting common commensal flora these specimens had a 75% positivity rate (*n* = 30) accounting for 16 distinct bacterial species. The most prevalent species isolated was *Staphylococcus aureus* (*n* = 5). While relatively common nosocomial pathogens such as *Enterococcus faecalis* (*n* = 2) were identified, so too were more unusual pathogens such as *Morganella morganii* (*n* = 2), *Enterobacter cloacae* (*n* = 3), and *Pseudomonas putida* (*n* = 1). Gram-negative species accounted for the majority of those identified (61%) and over half (*n* = 16) of the total isolates. The most common gram-negative bacteria encountered were *Enterobacter cloacae* (*n* = 3), *Klebsiella oxytoca* (*n* = 2), *Morganella morganii* (*n* = 2), and *Escherichia coli* (*n* = 2). Geographically, no significant difference between country of procedure and positive culture rate, type of species identified, or antimicrobial resistance in organisms was identified.

### Treatment types received

The most frequently prescribed antibiotics to treat this cohort of patients were piperacillin-tazobactam (168 total doses), co-amoxiclav (128 doses), flucloxacillin (50 doses), and clindamycin (46 doses). Of the 16 species isolated, a number of isolates displayed resistance to the most frequently prescribed empirical antibiotics such as co-amoxiclav (*n* = 4) and piperacillin-tazobactam (*n* = 6). In 2 cases, antibiotic treatment was changed based on culture results and antimicrobial resistance to an empirically prescribed antibiotic. Notably, four isolates identified met the criteria for multidrug resistant organisms (MDRO), which are defined as being non-susceptible to ≥ 1 agent in ≥ 3 antimicrobial categories, including a Vancomycin Resistant Enterococcus (VRE) and an Extended Spectrum Beta Lactamase (ESBL) producing isolate. Tables 1 and 2 detail the resistance patterns of the gram-positive and gram-negative isolates respectively.

**Table 1:** Gram-positive isolates and antimicrobial sensitivities

	Glycopeptides	Lincosamides	Oxacillin
*Staphylococcus aureus* (*n* = 5)		S, R	S
*Enterococcus faecalis* (*n* = 2)			
*Finegoldia magnia* (*n* = 2)			
*Staphylococcus lugdunensis* (*n* = 2)			
*Enterococcus faecium* (*n* = 2)	S, R		
*Staphylococcus warneri* (*n* = 1)			

*S*, sensitive; *R*, resistant; *NT*, not tested

**Table 2:** Gram-negative isolates and antimicrobial sensitivities

	Aminoglycosides	Carbapenems	Quinolones	3rd and 4th gen cephalosporins	Antipseudomonal penicillins + beta-lactamase inhibitors
*Enterobacter cloacae* (*n* = 3)			R	R	S, R
*Klebsiella oxytoca* (*n* = 2)			S		
*Morganella morganii* (*n* = 2)				S	R
*E coli* (*n* = 2)			R	S, R	R
*Pseudomonas aeruginosa* (*n* = 2)			S		S
*Citrobacter werkmanii* (*n* = 1)					S
*Pseudomonas putida* (*n* = 1)					S
*Klebsiella pneumoniae* (*n* = 1)	S		R		R
*Citrobacter koseri* (*n* = 1)	S				

*S*, sensitive; *R*, resistant; *NT*, not tested

Source control was required in 65% (*n* = 15) of patients admitted, with 10 patients requiring operative treatment and 7 interventional radiological procedures required.

A total of 76% (*n* = 23) of the patients required hospital admission with a mean length of stay of 6.65 days (range 1–20 days). Of the patients requiring admission, 78% (*n* = 18) required parenteral antibiotic treatment comprising a total of 181 days of therapy.

## Discussion

Our study has identified 35 separate infections occurring in 30 cosmetic tourists returning to Ireland and presenting to a regional plastic surgery center within the 15-month study period. The prevalence of cosmetic tourism, both domestically and internationally, has reportedly surged in recent years, the COVID-19 pandemic notwithstanding [1, 3, 6]. Several reasons have been proposed for this in the literature to date including lower costs to patients, lengthy waiting lists, availability of procedures not available in the public health service, and patient perception that quality of care is greater in cosmetic tourism destinations [1, 8]. This phenomenon has been widely reported internationally such as the UK and USA; however, a dearth of reported data exists for the Irish population [3]. Irish studies to date have focused on the cost burden to our health system of patients presenting with complications of primarily bariatric procedures carried out abroad [3, 19]. There is a paucity of available data in Europe regarding the microbiological etiology of infectious complications experienced by patients who have participated in cosmetic tourism.

Our study has demonstrated that while infectious complications in this cohort were found to be largely due to commonly reported soft tissue pathogens; in addition, a significant number of infections were associated with gram-negative organisms which may be due to a number of reasons including poor surgical technique and operative facilities, suboptimal wound care post-operatively, inappropriate antimicrobial prophylaxis, and delayed presentation to Irish health services with post-operative complications. Furthermore, the identification of multiple MDROs in our cohort is a cause for concern; for the potential problems they pose for treatment and the infection prevention and control issues they create. The presence of multiple concurrent infections in patients who had undergone more than one procedure was also found. This highlights the importance of both a thorough history and examination, comprising of appropriate wound samples from all relevant areas where appropriate. Additionally, early consultation with Clinical Microbiology in the context of the patient presentation and history should be sought, particularly in cases where concerns regarding response to empiric therapy are found. Plastic surgery units may need to consider revision of their empiric antimicrobial guidelines in some settings to account for the returning surgical patient.

The majority of literature on the microbial flora of medical tourism–related infections has been conducted in the USA where rapidly growing mycobacteria strains such as *Mycobacterium abscessus* have accounted for a significant proportion of cases [9, 13]. This finding was not identified in our study. The patient demographics in our study are consistent with existing literature, with patients most commonly female, in their 5th decade. The predominantly infectious nature of the complications we experienced was consistent both with Irish studies and those published abroad [1, 3, 9]. Authors attributed this difference in microbial etiology due to difference in geographical locations; cosmetic tourists in North America either avail of intra-bound tourism (within their home country) or in the Caribbean, including the Dominican Republic, where such organisms are more common [13].

While this study has a larger sample size than those hitherto published in this country, it is not without its limitations. The lack of published data in Europe on the microorganisms encountered in these infections makes it difficult to determine trends, either of wound flora or antimicrobial resistance. A further limitation, common to all studies on cosmetic tourism, is the fact that there are no reasonable or reliable means to record all patients from a country or region who partake of it, which makes contextualizing these complications challenging. Despite this, this study is the largest of its kind conducted in Ireland or the UK to date and may serve as a starting point for further studies on this cohort of patients.

Future studies on cosmetic tourism should, given the limited sample sizes and fragmented nature of studies to date, be conducted prospectively on a multi-center or national basis with standardized methodologies, protocols, and data points to allow detailed analysis of culture results, infectious complications, and possible changes required to empiric antimicrobial guidelines based on body site affected, type of procedure, and country of origin. Furthermore, studies involving direct comparison to non-travel related cosmetic infections would be of benefit. This would give a strong basis and inform any future population-level initiatives to mitigate the burden to our health service caused by these presentations.

## Conclusion

Cosmetic tourism is an increasingly common practice in Ireland and abroad. Its complications are most often infectious in nature and represent a considerable source of morbidity to patients. The presence of a wide range of pathogens, including antimicrobial-resistant organisms, may be considered in patients presenting with infections sustained as a result of cosmetic tourism and future large-scale prospective multi-center studies and surveillance projects are required to monitor and inform future approaches to patient management.

## References

[1] Miyagi K, Auberson D, Patel AJ, Malata CM (2012) The unwritten price of cosmetic tourism: an observational study and cost analysis. J Plast Reconstr Aesthet Surg 65:22–28. 10.1016/j.bjps.2011.07.027 21865103 10.1016/j.bjps.2011.07.027

[2] Jeevan R, Armstrong A (2008) Cosmetic tourism and the burden on the NHS. J Plast Reconstr Aesthet Surg 61:1423–1424. 10.1016/j.bjps.2008.10.002 18996337 10.1016/j.bjps.2008.10.002

[3] Rafeh S, Tara MC, Michael F et al (2022) An analysis of the cost and impact of cosmetic tourism and its associated complications: a multi institutional study. Surgeon 20:339–344. 10.1016/j.surge.2021.12.007 35012867 10.1016/j.surge.2021.12.007

[4] Murphy D, Lane-O’Neill B, Dempsey MP (2022) COVID-19 and cosmetic tourism: a Google trends analysis of public interests and the experience from a tertiary plastic surgery centre. J Plast Reconstr Aesthet Surg 75:1497–1520. 10.1016/j.bjps.2022.01.040 35148980 10.1016/j.bjps.2022.01.040

[5] Venditto C, Gallagher M, Hettinger P et al (2021) Complications of cosmetic surgery tourism: case series and cost analysis. Aesthet Surg J 41:627–634. 10.1093/asj/sjaa092 32291444 10.1093/asj/sjaa092

[6] Padilla P, Ly P, Dillard R et al (2018) Medical tourism and postoperative infections: a systematic literature review of causative organisms and empiric treatment. Plast Reconstr Surg 142:1644–1651. 10.1097/prs.0000000000005014 30489537 10.1097/PRS.0000000000005014

[7] McCrossan S, Martin S, Hill C (2021) Medical tourism in aesthetic breast surgery: a systematic review. Aesthetic Plast Surg 45:1895–1909. 10.1007/s00266-021-02251-1 33876284 10.1007/s00266-021-02251-1 PMC8054849

[8] Asher CM, Fleet M, Jivraj B, Bystrzonowski N (2020) Cosmetic tourism: a costly filler within the national health service budget or a missed financial opportunity? A local cost analysis and examination of the literature. Aesthetic Plast Surg 44:586–594. 10.1007/s00266-019-01571-7 31832735 10.1007/s00266-019-01571-7

[9] Cai SS, Chopra K, Lifchez SD (2016) Management of Mycobacterium abscessus infection after medical tourism in cosmetic surgery and a review of literature. Ann Plast Surg 77:678–682. 10.1097/sap.0000000000000745 26835829 10.1097/SAP.0000000000000745

[10] Varma P, Kiely J, Giblin AV (2022) Cosmetic tourism during the COVID-19 pandemic: dealing with the aftermath. J Plast Reconstr Aesthet Surg 75:506–508. 10.1016/j.bjps.2021.11.013 34838496 10.1016/j.bjps.2021.11.013 PMC8590619

[11] British Association of Plastic RaAS Cosmetic Surgery Abroad. Accessed 03/04/2024 2024

[12] Klein HJ, Simic D, Fuchs N et al (2017) Complications after cosmetic surgery tourism. Aesthet Surg J 37:474–482. 10.1093/asj/sjw198 28364525 10.1093/asj/sjw198

[13] Chen LH, Wilson ME (2013) The globalization of healthcare: implications of medical tourism for the infectious disease clinician. Clin Infect Dis 57:1752–1759. 10.1093/cid/cit540 23943826 10.1093/cid/cit540 PMC7107947

[14] Rogers BA, Aminzadeh Z, Hayashi Y, Paterson DL (2011) Country-to-country transfer of patients and the risk of multi-resistant bacterial infection. Clin Infect Dis 53:49–56. 10.1093/cid/cir273 21653302 10.1093/cid/cir273

[15] Ovadja ZN, Sluijmer H, Moerman E et al (2018) Rapidly growing mycobacteria infections among “cosmetic tourists” returning to the Netherlands. J Plast Reconstr Aesthet Surg 71:265–267. 10.1016/j.bjps.2017.10.023 29175134 10.1016/j.bjps.2017.10.023

[16] Robinson PD, Vaughan S, Missaghi B et al (2022) A case series of infectious complications in medical tourists requiring hospital admission or outpatient home parenteral therapy. J Assoc Med Microbiol Infect Dis Can 7:64–74. 10.3138/jammi-2021-0015 36340853 10.3138/jammi-2021-0015 PMC9603019

[17] Jabbour SF, Malek AE, Kechichian EG et al (2020) Nontuberculous mycobacterial infections after cosmetic procedures: a systematic review and management algorithm. Dermatol Surg 46:116–121. 10.1097/dss.0000000000001929 30964788 10.1097/DSS.0000000000001929

[18] Berríos-Torres SI, Umscheid CA, Bratzler DW et al (2017) Centers for disease control and prevention guideline for the prevention of surgical site infection, 2017. JAMA Surg 152:784–791. 10.1001/jamasurg.2017.0904 28467526 10.1001/jamasurg.2017.0904

[19] Dhannoon A, Atukoralalage U, Dhannoon A et al (2023) Challenges associated with global bariatric medical tourism. Ir Med J 116:809 37606236

